# Detection and Identification of *Toxoplasma gondii* Type One Infection in Sheep Aborted Fetuses in Qazvin Province of Iran

**Published:** 2012

**Authors:** GR Habibi, AR Imani, MR Gholami, MH Hablolvarid, AM Behroozikhah, M Lotfi, M Kamalzade, E Najjar, K Esmaeil-Nia, S Bozorgi

**Affiliations:** 1 Parasite Vaccine Research and Production Department, Razi Vaccine and Serum Research Institute, Karaj, Iran; 2 Veterinary College, Islamic Azad University, Karaj, Iran; 3 Pathology Department, Razi Vaccine and Serum Research Institute, Karaj, Iran; 4 Brucellosis Vaccine Research and Production Department, Razi Vaccine and Serum Research Institute, Karaj, Iran; 5 Quality Control of Biological Products group, Razi Vaccine and Serum Research Institute, Karaj, Iran; 6 Veterinary Organization of Qazvin Province, Qazvin, Iran

**Keywords:** *Toxoplasma gondii*, Sheep abortion, Fetus, PCR, Bioassay, Genotyping

## Abstract

**Background:**

The aim of this study was to apply the nested-PCR and bioassay methods in detection and genotyping of *Toxoplasma gondii* infection in provided sheep aborted fetus samples from Qazvin Province of Iran.

**Methods:**

Eighteen sheep aborted fetal samples were studied by nested-PCR-RFLP, histopathological observation and microbiological assay. Bioassay in mice was carried out by inoculating the brain samples intraperitoneally.

**Results:**

The results demonstrated the frequency of 66% infected sheep aborted fetal samples with *T. gondii* type one. Although we could not isolate any parasite from inoculated mice even after three passages, but it was confirmed histopathologically formation of cyst like bodies in prepared mice brain sections.

**Conclusion:**

The results of the performed nested-PCR and formation of brain cyst in inoculated mice exhibited that *T. gondii* type one infection might be considered as one of the major causative agents for abortion in ewes.

## Introduction

Toxoplasmosis is a parasitic disease caused by the protozoan *Toxoplasma gondii*. It has worldwide distribution (1). *Toxoplasma gondii* is an important zoonotic pathogen and a major cause of reproductive failure associated with abortion in sheep and goat (2, 3). In sheep, fetal resorption, abortion or prenatal mortality of lambs occur when ewes suffer a primary infection during pregnancy. Infected lamb is considered the main source of toxoplasmosis worldwide (3).

The diagnosis of *T. gondii* infection is usually based on histopathological examination, serological assay, and isolation of *Toxoplasma* by mouse inoculation (4–7). Most of epidemiological studies on *T. gondii* have been done based on extensive serological tests in all over the world including Iran. The seroprevalence of toxoplasmosis were reported between 13.8% and 35% for sheep, 13.1 and 30% for goats, 0% and 16% for cattle, 4.7% and 10.8% for buffaloes and 11.5% for horses in different parts of Iran (8–13). Molecular assays, such as the PCR make it possible to detect small quantities of target DNA and potentially provide an alternative sensitive diagnostic tool (4, 14).

In this study, fetal samples were used to assay from sheep aborted fetuses. The B1 gene as a target sequence using PCR-RFLP was applied for detection and genotyping the *T*. *gondii*. The aim of this study was to apply the nested-PCR and bioassay methods in detection and genotyping of *T. gondii* infection in provided sheep aborted fetus samples from Qazvin Province of Iran.

## Materials and Methods

The biological samples were collected from eighteen sheep aborted fetuses in Qazvin province, central Iran. The samples were transported under refrigeration to parasitic vaccine research and production laboratory of Razi Institute, Karaj, Iran where they were analyzed. Firstly, the brain of each fetus was removed from lamb skull aseptically for direct parasite detection. The prepared brain tissue samples were kept frozen for further DNA extraction, PCR assay, mice inoculation for bioassay, histopathological observation, and microbiological examination.

Bioassay in mice was accomplished based on OIE instructions (15). Briefly, 20–30 g of brain was collected, homogenized by using a tissue homogenizer (Seward, Stomacher 400, England) in sterile PBS and then was filtered through two layers of gauze and centrifuged for 10 min at 2000 g. Pellet was re-suspended in 7 ml of PBS, and two aliquots prepared. One aliquot was frozen and stored at -20 °C until PCR was performed; the other was inoculated intraperitoneally into five female *T. gondii*-free BALB/c and NMRI mice (Razi institute, Laboratory Animals Production Dept.).

Mice were observed for up to 45 days. Samples of lung, liver, spleen, and brain were collected from mice that died during the observation period for cytological analysis, and were stained by Giemsa. Attempts were made to isolate and propagate the protozoa, by intraperitoneal inoculation of three week-old BALB/c and NMRI mice (16). Peritoneal fluids were examined from mice inoculated with brain suspensions of sheep aborted fetuses, and were used in three blind passages, for detection of the organism.

*Toxoplasma gondii* strain RH (type I) was used for bioassay standardization and as positive control of PCR assays (17, 18). Parasites were propagated and maintained in susceptible mice and cell culture. The virulent RH strain of *T. gondii* was maintained by intraperitoneal passages in 6-8-week old, male BALB/c and NMRI mice. The mice were sacrificed after 3-4 days by ether inhalation. Tachyzoites were harvested from mice on the third day of infection by lavage of the peritoneal cavity with 5 ml of RPMI 1640 medium (Sigma, St. Louis, USA), containing a mixture of 100 U/ml penicillin and 100 µg/ml streptomycin (Biosera, Sussex, UK) (18).

The Vero cell line used as host cell was maintained with RPMI-1640, in cell culture flasks and was incubated at 37°C in a 5% CO_2_ atmosphere. Vero cells were obtained from the Virology section of quality control department of Razi institute, were grown in 25 Cm^2^ flasks (NUNC, Roskilde, Denmark) in 10 ml of culture medium: RPMI-1640 medium with supplemented with L-glutamine (2 mM) (Merck, Germany), Penicillin-Streptomycin Solution 100X (frozen) (Biosera, Sussex, UK), and foetal calf serum (FCS) (Biosera, South America) at concentrations of 10% and 2%. The cells were grown in RPMI-1640 with 10% FCS (growth medium) at 37 °C in sealed flasks. When a confluent monolayer was obtained, the medium was changed to RPMI-1640 with 2% FCS (maintenance medium).

The original inocula of *Toxoplasma gondii* RH strain were peritoneal exudates from infected BALB/c mice (18). Parasite inoculum in 100 µl infection medium was added to each flask. The infection was allowed for 16 h at 37 °C as described previously (5). The medium of infected culture was removed and the cells were washed twice with maintenance medium. The cultures were checked by inspection under an inverted microscope to verify the presence of released tachyzoites.

DNA extraction was performed using DNA digestion buffer (0.5% SDS, 25 mM EDTA, 100 mM NaCl, 20 mM Tris-HCl (pH 8.0), and proteinase K [0.1 mg/ml final concentration]). Digested tissues were extracted with phenol chloroform isoamyl alcohol (25:24:1), and the DNA was precipitated in 0.3 M sodium acetate (final concentration) with 2 volumes of pure cold ethanol. DNA was solubilized in TE buffer (10 mM Tris-HCl, 1mM EDTA) and stored at -20 °C. The DNA concentration was estimated by UV spectrophotometric absorbance at 260 nm (19).

Specific primers were selected to amplify a portion of B1 gene for identification and further genotype analysis. The specific *T. gondii* primers for 35-fold repeated B1 gene sequence (GenBank accession no. AF179871) were selected to amplify a 580 bp DNA fragment by external primer pair; Tg1 (5’ TGT TCT GTC CTA TCG CAA CG) and Tg2 (5’ ACG GAT GCA GTT CCT TTC TG) in the first round of PCR. The internal primer sequences were Tg3 (5’ TCT TCC CAG ACG TGG ATT TC) and Tg4 (5’ CTC GAC AAT ACG CTG CTT GA) to amplify a 531 bp DNA fragment in a secondary step (nested-PCR) (20). PCR amplification was carried out on genomic DNA. Each PCR utilized 2.5 µl of PCR buffer (10X PCR buffer), 15 mM MgCl_2_, 0.1 mM dNTPs, 10 pmole of each primer, and 0.5 U of Taq DNA polymerase in a total reaction volume of 25 µl. PCR were performed in a thermocycler (Techne, Techgen, Germany) for 35 cycles of denaturation at 94 °C for 15S, annealing at 60 °C for 45S and extension at 72 °C for 45S. A nested primers optimized to PCR amplify B1 DNA to permit sensitive detection of parasite in clinical and experimental samples. The amplicons were 1:100 diluted in deionized distilled water and then used for nested PCR with internal primers Tg3 and Tg4. PCR products were visualized using on ethidium bromide stained 1.5% agarose gel.

Genetic characterization of isolates was performed by the PCR-RFLP method described by Grigg and Boothroyd (20). There is a restriction site into amplified DNA, XhoI (C/TCGAG) restriction site (nucleotide positions 366) fall within potential restriction site for XhoI, in type II and III sequences. The restriction fragments were resolved in 2% agarose gel, stained with ethidium bromide and photographed using a gel documentation system (Uvitec, Cambridge UK).

**Fig. 1:**
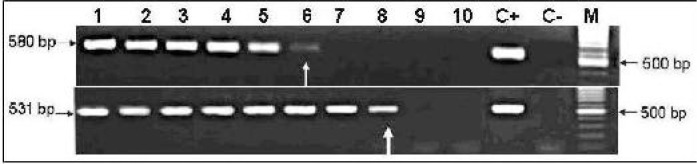
Determination of sensitivity of the nested-PCR for detecting *Toxoplasma gondii* B1 gene. Extracted *T. gondii* infected DNA was quantified, then serial dilutions were prepared, followed by the first and second round of PCR. Lanes 1 to 10 exhibits the 10^0^ to 10^-9^ dilutions of infected DNA; lane C+ is positive control and lane C- is negative control. The results showed the sensitivity for the 1^st^ round of PCR was 17 pg/µl *Toxoplasma gondii* infected DNA (lane 6), and 170 fg/µl infected DNA (lane 8) for the nested-PCR. Lane M shows the 100 bp DNA size marker. Left black arrows show the size of PCR products for 1^st^ and 2^nd^ PCR products.

The sensitivity of the PCR was determined by preparation of serial dilutions from a *T. gondii* infected DNA sample and then was subjected to the nested-PCR for gene B1. The infected Vero cell line with tachyzoite of *T. gondii* RH strain was used for DNA extraction and quantification. Then, DNA solution was diluted (neat to 10^-9^) and PCR was carried out by external primers and the amplicon of the first PCR was used for the second round of PCR by using internal primers (nested-PCR) (Fig. 1).

PCR specificity was shown by using some apicomplexan DNA, including *Neospora caninum*, *Sarcocystis* spp., *Babesia ovis*, *Theileria annulata*, and healthy sheep genomic DNA as well as negative control (data not shown). In addition, specificity of the PCR was verified by restriction analysis and PCR product nucleic acid sequencing.

The amplicons of gene B1 obtained from studied *T. gondii* infected samples were purified and were sent for bi-directional nucleic acid sequencing (MWG-Biotech, Germany). The received data of DNA sequencing were aligned with available vast sequences in GenBank by “Blast n” online program (http://blast.ncbi.nlm.nih.gov/Blast.cgi).

Brain tissue samples were provided from sheep aborted fetuses and inoculated to mice, were fixed in 10% formalin. The fixed brain tissues were sectioned and stained with Hematoxylin-Eosin (H and E) for histopathological examinations (21, 22). Moreover, mice brain samples were sent for histopathological examination for possible *T. gondii* infection.

Bacteriological tests were conducted for causative agents of abortion in sheep; *Brucella* spp., *Campylobacter* spp., *Salmonella* spp., and fungal agents in all sheep aborted fetal samples (23, 24).

## Results

The standard RH strain was subjected to bioassay in mice. The experimental infection has been determined by showing the tachyzoite of *T. gondii* (RH strain) from ascitic fluids of inoculated BALB/c and NMRI mice. The parasite was grown and propagated in Vero cell culture in appropriate media and has been seen by microscopical observation.

Twelve samples out of eighteen sheep aborted fetal specimens were positive in specific *T. gondii* nested PCR assay.

The positive samples were checked for B1 gene sequencing and the analyzed *T. gondii* B1 gene sequences were verified the PCR specificity. In addition, the sequencing information was aligned by "Blast n" and the results were exhibited 96 to 100% identity to *T. gondii* B1 sequences deposited in GenBank. The obtained local *T. gondii* B1 gene sequence was registered in GenBank (acc. no. DQ789361).

The limit of detection was determined for at least 17 pg/µl of *T. gondii* infected DNA for the first PCR, but this border was decreased to 170 fg/µl of DNA in the second round of PCR (nested reaction) (Fig. 1).

*In silico* analysis of selected primers for *T. gondii* B1 gene sequence demonstrated all of them are specific for *T. gondii* B1 gene sequences deposited in GenBank and exhibited 100 percent homology. For *in vitro* study, the selected *T. gondii* B1 gene primers were used to amplify *T. gondii* RH DNA and other available apicomplexan protozoa DNAs, including *Babesia ovis*, *Theileria* spp., *Neospora caninum*, *Sarcocystis* spp., and healthy sheep genomic DNA as well as negative control. The results showed the primers were specific for *T. gondii* and no amplification was seen by other used DNAs.

**Fig. 2:**
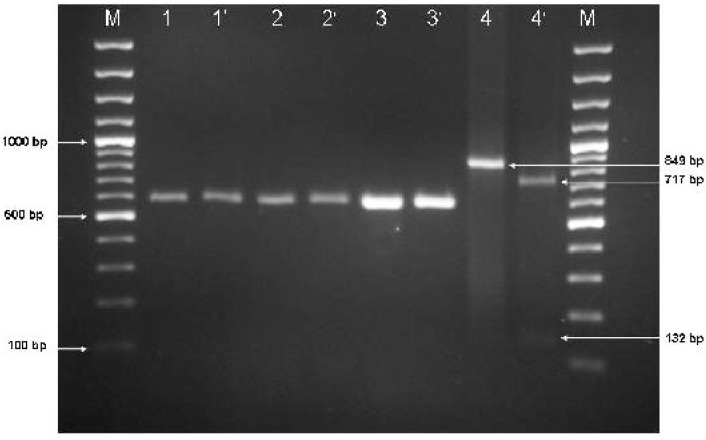
Gel agarose electrophoresis (2%) for *Toxoplasma gondii* PCR products and restriction enzyme analysis Lane 1 to 6: *Toxoplasma gondii* B1 gene PCR products (580 bp) were amplified by external primers. M: 100 bp DNA size marker. Lanes 1 and 2 are two positive clinical samples for *T. gondii* B1 gene before digestion and lanes 1’ and 2’ are after digestion by XhoI restriction enzyme. Lanes 3 and 3’ are control positive *T. gondii* RH strain type I before and after restriction enzyme treatment respectively. Lanes 4 and 4’ are showing the positive DNA (has a cut site for XhoI) before and after digestion respectively, lane 4 shows the undigested band of 849 bp length, and lane 4’ reveals two digested bands of 717 and 132 bp. Size of PCR products and digested products were pointed.

The pattern of digestion of the reference RH strain was matched to the expected DNA fragments. The RH strain was characterized as genotype I because the amplified fragment of the B1 gene was undigested with the corresponding restriction enzyme. Genotyping of the twelve-studied positive sample for *T. gondii* determined the all available samples are type I. The positive DNA fragment having a cut site for Xho I restriction enzyme, was digested as a positive control for the enzyme activity. Figure 2 shows the digestion pattern of two *T. gondii* positive samples.

**Fig. 3:**
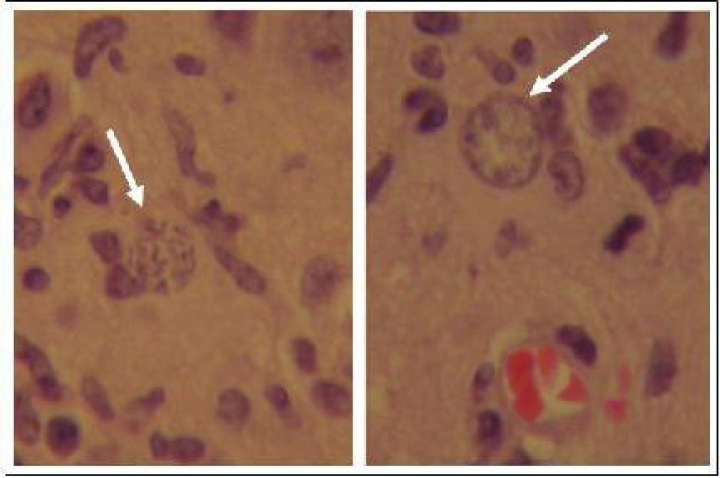
Micrographs of mouse brain section stained by hematoxylin and eosin, demonstrates the *Toxoplasma gondii* cyst (arrows) formation after three passages of sheep aborted fetal brain samples inoculated through IP in NMRI mice (1000X)

There was no symptom of *T. gondii* infection in histopathological assay on sheep aborted fetal sections, but in mice brain sections, some signs of CNS involvement were observed including cyst-forming bradyzoite, encephalitis, and perivascular cuffing (PVC) (Fig. 3).

The microbiological assays have revealed, the five out of eighteen sheep aborted fetal samples were infected by *Brucella melitensis* biotype I, but no other causative bacterial or fungal agents for abortion were detected in our study.

## Discussion

The protozoan *T. gondii* is an obligate intracellular parasite in human being and agricultural animals worldwide (1). Ingesting tissue cysts from undercooked meat, consuming food, or drink contaminated with oocysts, or by accidentally ingesting oocysts from the environment are the ways for human infection. However, the ingestion of undercooked infected lamb is considered an important source of infection for human particularly pregnant women (25). Aborted fetuses to be included in the current study were selected as the specimens for detection and isolation of causative agents for abortion in sheep particularly *T*. *gondii*. Different methods were performed for detecting *T. gondii* infection, including molecular methods, *in vitro* and *in vivo* bioassay in cell tissue culture and mice inoculation respectively, histopathological and microbiological examinations.

The main purpose of this study was to perform a specific and sensitive PCR assay to detect *T. gondii* in sheep aborted fetal samples. Moreover, both histopathology and microbiology techniques were used to broad this diagnostic investigation. The PCR assay was based on the *T. gondii* B1 gene sequence. B1 gene is a tandemly arrayed 35 fold repetitive gene routinely used for this highly specific and sensitive PCR detection of *T. gondii* present in clinical specimens (26–29).

Genetic studies have identified three distinct groups type I, mouse virulent; types II and III are two mouse non-virulent strains (4). It is unclear whether a similar situation exists for sheep infection. Since infection in pregnancy may cause severe damage to the fetus, a positive PCR result was derived from small tissue samples is expected to improve the prognosis because control and preventive program can be initiated when the footprint of the parasite have been shown in examined specimens. Nested-PCR gives valuable evidences that support the choice of specific prevention for animals those exposed to several pathogens (30).

Asgari et al. have performed a molecular study of samples were taken from different organs of sheep and goats. The results of PCR indicated the prevalence of *T. gondii* infection was found to be 22.7% for goats and 37.5% for sheep. The highest infected tissue was determined 21.8% for tongue followed by brain (19.2%) and femoral and intercostal muscles (17.9%). These findings emphasized a high level of *Toxoplasma* infection in slaughtered animals in Shiraz and might be considered as the main sources of infection for human (31).

Razmi et al. showed the role of *T. gondii* in inducing sheep abortion in Mashhad area of Iran by using serological and parasitological methods. The seropositive ovine fetuses were found 5.2% of 325 ovine fetuses. They concluded that *T. gondii* is one of the most important causes of ovine abortion in Iran (32).

Zia-Ali et al. (33) have reported the isolation of four *T. gondii* from adult sheep in Iran, two isolates were type II and two were type III. In summary, previously published data indicated that genotypes II are the predominant strains in sheep, but, no *T. gondii* type I isolate has been reported from sheep so far (34). However, interestingly, our data clearly indicated all positive *T. gondii* isolates from sheep aborted fetal samples in Qazvin Province were genotype I. There are several possible explanations for these differences in observed *T. gondii* genotypes, an effect of differential selection of the strains, the epidemiological prevalence of the parasites and finally the route of transmission may be very different in regions, where studies were performed (35).

Besides the using cell culture or mice inoculation to isolate and growing the parasite from clinical samples, the results of this study demonstrate that molecular epidemiological studies on *T. gondii* infection may be performed directly on infected tissue samples. The nested PCR followed by RFLP assay used here is a fast and a highly sensitive method for detecting *Toxoplasma* infection and characterizing parasite genotype directly on clinical samples in comparison with time-consuming techniques required to grow the parasite and possible loss of samples or strain selection during culture and or mice inoculation (20, 35, 36).

The prevalence of infectious parasites and adequate live infective parasite are the most important parameters for mouse infection. Although the isolation of the parasite in mice is considered the "gold standard," but the technique is cumbersome and time-consuming, and success is impacted by variable susceptibility of the mice, virulence of the infecting parasite, the route and dose of the inoculum as well as the content of potentially viable parasite (37). The demonstration of *Toxoplasma* infection in mice is often delayed since mice usually develop a chronic rather than an acute infection (38).

In this study, we started with fetal samples from the Qazvin veterinary laboratory. The aborted fetuses have been brought from the field and have been remained in fridge at laboratory for one day. Therefore, we have to process the samples in our lab when fetuses were kept in 4°C for near two days. The possibility of fetal tissues lyses and probable contamination could not be ignored.

Interestingly, the observed histopathological findings mentioned previously showed the microscopic symptoms of Toxoplasmosis particularly cyst formation in the prepared brain sections against the confirmed *T. gondii* type I infection by PCR-RFLP. Moreover, all inoculated mice had no clinical symptoms of toxoplasmosis and were killed after four months for histopathological examination. Therefore, the confirmation of *T. gondii* type one infection in inoculated mice with low virulence characteristic, creates this hypothesis that assigning the very virulent could not be generalized to all *T. gondii* type one isolates /strains.

According to the positive results in microbiological assays, *B. mellitensis* was isolated from studied samples. These findings explain the importance of *Brucella* as a potential cause of abortion in ewes in Iran (23, 24). The four out of five positive *B. mellitensis* infected samples were co-infected with *T. gondii* as well. This emphasizes again for importance of these two potentially infective agents in abortion induction.

However, the finding of infective agent or seropositive titer in studied samples cannot necessarily prove the cause of abortion. Therefore, the actual cause might be estimated with an analysis of the various reasons for abortion, with emphasis on pathogen levels in the samples to be investigated.

In conclusion, this investigation provides a new data for understanding the role of ovine in epidemiology of *T. gondii* for using it in possible relationship between parasite genotypes and severity of toxoplasmosis in sheep and human. Finding the nutrition pattern of a region, detection of *T. gondii* infection in lamb, severity of toxoplasmosis, and circulating genotypes in the nature all might be helpful for showing the association between toxoplasmosis in humans and sheep for disease control as well as preventive actions.

## References

[1] Dubey JP, Beattie CP (1988). Toxoplasmosis of animals and man.

[2] Buxton D (1990). Ovine toxoplasmosis: a review. J R Soc Med..

[3] Tenter AM, Heckeroth AR, Weiss LM (2000). *Toxoplasma gondii*: from animals to humans. Int J Parasitol..

[4] Remington JS, Thulliez P, Montoya JG (2004). Recent developments for diagnosis of toxoplasmosis. J Clin Microbiol..

[5] Garcia JL, Gennari SM, Machado RZ, Navarro IT (2006). *Toxoplasma gondii*: detection by mouse bioassay, histopathology, and polymerase chain reaction in tissues from experimentally infected pigs. Exp Parasitol..

[6] da Silva AV, Langoni H (2001). The detection of *Toxoplasma gondii* by comparing cytology, histopathology, bioassay in mice, and the polymerase chain reaction (PCR). Vet Parasitol..

[7] Masala G, Porcu R, Madau L, Tanda A, Ibba B, Satta G, Tola S (2003). Survey of ovine and caprine toxoplasmosis by IFAT and PCR assays in Sardinia, Italy. Vet Parasitol..

[8] Ghorbani M, Hafizi A, Shegerfcar MT, Rezaian M, Nadim A, Anwar M, Afshar A (1983). Animal toxoplasmosis in Iran. J Trop Med Hyg..

[9] Hoghooghi-Rad N, Afraa M (1993). Prevalence of toxoplasmosis in humans and domestic animals in Ahwaz, capital of Khuzestan Province, south-west Iran. J Trop Med Hyg..

[10] Hashemi-Fesharki R (1996). Seroprevalence of *Toxoplasma gondii* in cattle, sheep and goats in Iran. Vet Parasitol..

[11] Navidpour S, Hoghooghi-Rad N (1998). Seroprevalence of anti-*Toxoplasma gondii* antibodies in buffaloes in Khuzestan province, Iran. Vet Parasitol..

[12] Sharif M, Gholami Sh, Ziaei H, Daryani A, Laktarashi B, Ziapour SP, Rafiei A, Vahedi M (2007). Seroprevalence of *Toxoplasma gondii* in cattle, sheep and goats slaughtered for food in Mazandaran province, Iran, during 2005. Vet J..

[13] Ghazaei C (2006). Serological survey of antibodies to Toxoplasma gondii. Afr J Health Sci..

[14] Müller N, Zimmermann V, Hentrich B, Gottstein B (1996). Diagnosis of *Neospora caninum* and *Toxoplasma gondii* infection by PCR and DNA hybridization immunoassay. J Clin Microbiol..

[15] OIE Terrestrial Manual 2008 http://www.oie.int/fileadmin/Home/eng/Health…/2.09.10_TOXO.pdf.

[16] Masihi KN, Werner H (1977). Immunization of NMRI mice against virulent *Toxoplasma gondii*. Differing efficacy of eleven cyst-forming *Toxoplasma* strains. Z Parasitenkd..

[17] Dubey JP, Shen SK, Kwok OC, Frenkel JK (1999). Infection and immunity with the RH strain of *Toxoplasma gondii* in rats and mice. J Parasitol..

[18] Evans R, Chatterton JMW, Ashburn D, Joss AWL, Ho-Yen DO (1999). Cell-Culture System for Continuous Production of *Toxoplasma gondii* Tachyzoites. Eur J Clin Microbiol Infect Dis..

[19] Sambrook J, Fritsch EF, Maniatis T (1989). Molecular Cloning: A Laboratory Manual.

[20] Grigg ME, Boothroyd JC (2001). Rapid identification of virulent type I strains of the protozoan pathogen *Toxoplasma gondii* by PCR-restriction fragment length polymorphism analysis at the B1 gene. J Clin Microbiol..

[21] Handan A (1992). The significance of histopathological diagnosis of toxoplasmosis. Turk J Med Res.

[22] Esteban-Redondo I, Maley SW, Thomson K, Nicoll S, Wright S, Buxton D, Innes EA (1999). Detection of *T. gondii* in tissues of sheep and cattle following oral infection. Vet Parasitol..

[23] Brucellosis in Sheep and Goats *Brucella melitensis* (2001). European Commission Health and Consumer Protection Directorate-general Directorate C - Scientific HealthOpinions. http://ec.europa.eu/food/fs/sc/scah/out59_en.pdf.

[24] Sharifiyazdi H, Haghkhah M, Behroozikhah AM, Nematgorgani E (2010). Bacteriological and molecular investigation of *B. melitensis* in dairy cows in Iran.

[25] Cook AJ, Gilbert RE, Buffolano W, Zufferey J, Petersen E, Jenum PA, Foulon W, Semprini AE, Dunn DT (2000). Sources of *Toxoplasma* infection in pregnant women: European multicentre case-control study. European Research Network on Congenital Toxoplasmosis. BMJ..

[26] Chumpitazi BF, Boussaid A, Pelloux H, Racinet C, Bost M, Goullier-Fleuret A (1995). Diagnosis of congenital toxoplasmosis by immunoblotting and relationship with other methods. J Clin Microbiol..

[27] Filisetti D, Gorcii M, Pernot-Marino E, Villard O, Candolfi E (2003). Diagnosis of congenital toxoplasmosis: comparison of targets for detection of *Toxoplasma gondii* by PCR. J Clin Microbiol..

[28] Homan WL, Vercammen M, De Braekeleer J, Verschueren H (2000). Identification of a 200- to 300-fold repetitive 529 bp DNA fragment in *Toxoplasma gondii*, and its use for diagnostic and quantitative PCR. Int J Parasitol..

[29] Liesenfeld O, Montoya JG, Kinney S, Press C, Remington JS (2001). Effect of testing for IgG avidity in the diagnosis of *Toxoplasma gondii* infection in pregnant women: experience in a US reference laboratory. J Infect Dis..

[30] Montoya A, Miró G, Mateo M, Ramírez C, Fuentes I (2009). Detection of *Toxoplasma gondii* in cats by comparing bioassay in mice and polymerase chain reaction (PCR). Vet Parasitol..

[31] Asgari Q, Sarnevesht J, Kalantari M, Sadat SJ, Motazedian MH, Sarkari B (2011). Molecular survey of Toxoplasma infection in sheep and goat from Fars province, Southern Iran. Trop Anim Health Prod..

[32] Razmi GR, Ghezi K, Mahooti A, Naseri Z (2010). A serological study and subsequent isolation of *Toxoplasma gondii* from aborted ovine fetuses in Mashhad area, Iran. J Parasitol..

[33] Zia-Ali N, Fazaeli A, Khoramizadeh M, Ajzenberg D, Darde′ M, Keshavarz-Valian H (2007). Isolation and molecular characterization of *Toxoplasma gondii* strains from different hosts in Iran. Parasitol Res..

[34] Dubey JP (2009). Toxoplasmosis in sheep--the last 20 years. Vet Parasitol..

[35] Fuentes I, Rubio JM, Ramírez C, Alvar J (2001). Genotypic characterization of *Toxoplasma gondii* strains associated with human toxoplasmosis in Spain: direct analysis from clinical samples. J Clin Microbiol..

[36] Su C, Zhang X, Dubey JP (2006). Genotyping of *Toxoplasma gondii* by multilocus PCR-RFLP markers: a high resolution and simple method for identification of parasites. Int J Parasitol..

[37] Derouin F, Sarfati C, Beauvais B, Iliou M.-C, Dehen L, Lariviere M (1989). Laboratory diagnosis of pulmonary toxoplasmosis in patients with acquired immunodeficiency syndrome. J Clin Microbiol..

[38] Derouin F, Mazeron M C, Garin Y J (1987). Comparative study of tissue culture and mouse inoculation methods for demonstration of *Toxoplasma gondii*. J Clin Microbiol..

